# A Framework to Evaluate Feasibility, Safety, and Accuracy of Wireless Sensors in the Neonatal Intensive Care Unit: Oxygen Saturation Monitoring

**DOI:** 10.3390/s25185647

**Published:** 2025-09-10

**Authors:** Eva Senechal, Daniel Radeschi, Emily Jeanne, Ana Saveedra Ruiz, Brittany Dulmage, Wissam Shalish, Robert E. Kearney, Guilherme Sant’Anna

**Affiliations:** 1 Experimental Medicine Department, McGill University Health Center, Montreal, QC H3A 0G4, Canada; eva.senechal@mail.mcgill.ca (E.S.); emily.jeanne@mail.mcgill.ca (E.J.); 2 Biomedical Engineering Department, McGill University, Montreal, QC H3A 0G4, Canada; daniel.radeschi@mail.mcgill.ca (D.R.); robert.kearney@mcgill.ca (R.E.K.); 3 Research Institute of the McGill University Health Center, Montreal, QC H4A 3J1, Canada; ana.saavedra.ruiz@muhc.mcgill.c (A.S.R.); wissam.shalish@mcgill.ca (W.S.); 4 Wexner Medical Center, University of Ohio, Columbus, OH 43210, USA; brittany.dulmage@osumc.edu; 5 Neonatal Division, McGill University Health Center, Montreal, QC H4A 3H9, Canada

**Keywords:** NICU, wireless sensors, wearable devices, oxygen saturation

## Abstract

**Highlights:**

**What are the main findings?**

- The study introduces and applies a robust, multidimensional framework for assessing wireless monitoring devices in NICUs—addressing feasibility, safety, and clinical accuracy in real-world conditions.
- Applying this framework to a wireless oximeter revealed a strong performance for skin safety and Bluetooth connectivity of device but issues with signal coverage and accuracy.

**What is the implication of the main finding?**

- The study’s framework offers a standardized approach for evaluating emerging wireless technologies in neonatal care, encouraging global assessments of feasibility, safety and accuracy.
- The findings of this study suggest future development is needed to address remaining challenges in wireless oximeters, related to their accuracy and signal coverage.

**Abstract:**

Monitoring vital signs in the Neonatal Intensive Care Unit (NICU) typically relies on wired skin sensors, which can limit mobility, cause skin issues, and interfere with parent–infant bonding. Wireless sensors offer promising alternatives, but evaluations to date often emphasize accuracy alone, lack NICU-specific validation, and rarely use standardized frameworks. Our objective was to develop and apply a comprehensive framework for evaluating the feasibility, safety, and accuracy of wireless monitoring technologies using a wireless pulse oximeter, the Anne limb (Sibel Health, USA), in real-world NICU conditions. A prospective study was conducted on a diverse NICU population. A custom system enabled synchronized data recordings from both standard and wireless devices. Feasibility was assessed as signal coverage across a variety of daily care activities and during routine procedures. Safety was evaluated through skin assessments after extended wear. Accuracy was examined sample-by-sample and interpreted using the Clarke Error Grid for clinical relevance. The wireless oximeter device showed high feasibility with reliable Bluetooth connection across a range of patients and activities (median wireless PPG coverage = 100%, IQR: 99.85–100%). Skin assessments showed no significant adverse effects. Accuracy was strong overall (median bias 1.34%, 95% LoA −3.63 to 6.41), with most data points within clinically acceptable Clarke error grid zones A and B, though performance declined for infants on supplemental oxygen. This study presents a robust, multidimensional framework for evaluating wireless monitoring devices in NICUs and offers recommendations for future research design and reporting.

## 1. Introduction

Patients in the Neonatal Intensive Care Unit are at high risk of sudden physiological deterioration [1]. Therefore, continuous monitoring of vital signs is an essential part of care, allowing providers to closely monitor and rapidly respond to changes in patient status [2]. Routine monitoring of NICU patients typically relies on wired, adhesive sensors that pose several challenges [1,2,3,4,5]. These wires can interfere with infant positioning, become soiled or disconnected during care, and contribute to caregiver burden and parental distress bonding [6,7,8,9,10]. Additionally, the sensors generate heat and may cause burns and pressure sores, requiring frequent repositioning [7,11]. Despite these limitations, the core technology of bedside vital signs monitoring has remained largely unchanged since its clinical adoption in the 1980s [12,13].

In recent years, wireless monitoring technologies have emerged as a promising alternative to reduce the burden of wires and improve patient comfort and care workload. However, many of these devices were developed for home or commercial use, and clinical studies have raised significant accuracy concerns, limiting their applicability in the NICU [14,15,16]. Generally, studies assessing wireless vital sign sensors in NICU populations are limited in number and scope, often focusing narrowly on short-term accuracy rather than broader clinical performance [17]. The limited number of existing studies also demonstrate significant heterogeneity in methodology, with small sample sizes, brief recording durations, and limited reporting of outcomes related to feasibility and safety [17,18,19,20]. As such, there remains a need for a standardized approach to evaluate new wireless monitoring technologies in the NICU context.

In response to these gaps, as part of a large clinical study, we developed a framework to evaluate new wireless sensors in a real-world NICU setting. The aim of this study is to describe this structured framework and demonstrate its use to evaluate feasibility, safety, and accuracy of using the wireless pulse oximeter (SpO_2_) signal.

## 2. Materials and Methods

### 2.1. Primary Outcome

The primary outcome of this study is the development of a rigorous and reproducible methodological framework for the clinical testing and reporting of results of studies examining novel wireless vital sign sensors for the NICU. This framework included detailed methodology and systematic presentation of the results as outlined below. The SpO_2_ wireless signal was used for the detailed presentation of the framework.

### 2.2. Study Design

A prospective observational study was carried out at the NICU of the Montreal Children’s Hospital between August 2022 and March 2023. The study protocol was registered on ClinicalTrials.gov (NCT04956354) and has been previously published [21]. Ethical approval was granted by the McGill University Health Centre Research Ethics Board (approval #2022–7704). Informed written consent was obtained from the parents of all enrolled infants prior to participation.

### 2.3. Study Participants

Infants of any gestational age (GA) were eligible. Infants with fragile skin, congenital skin infections, or major congenital anomalies were excluded.

### 2.4. Study Equipment and Recording

Neonates in the NICU were monitored using the standard wired system (Philips Intellivue MX450, Philips, The Netherlands) in conjunction with a Masimo LCNS neonatal pulse oximeter (LCNS Neo, Masimo, Irvine, CA, USA). The photoplethysmographic (PPG) signal was nominally sampled at 75 Hz, while oxygen saturation (SpO_2_) values were recorded at 1 Hz using a standard fixed 8s averaging window.

**Figure 1:**
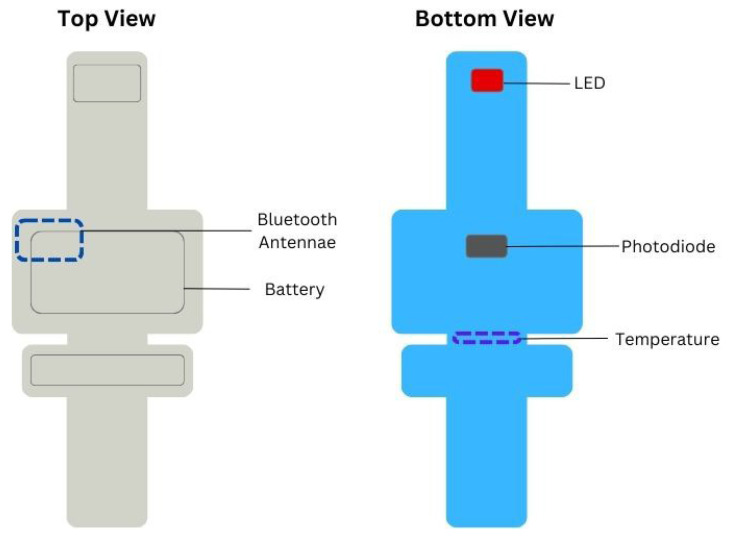
Wireless limb sensor. Legend: Design and internal components of the wireless limb sensor.

In parallel, a wireless sensor (ANNE^®^ Limb, Sibel Health Inc., Chicago, IL, USA) was secured to either the foot or hand with a disposable soft fabric strap. This limb unit included dual-wavelength red and infrared LEDs, a photodiode, a temperature sensor, battery, and Bluetooth antenna (Figure 1). The wireless PPG signal was sampled at 64 Hz, and SpO_2_ was also recorded at 1 Hz. Data were transmitted via Bluetooth to a research Android tablet running a custom-built Biosensors Data Aggregation and Synchronization (BioDAsh) app, which displayed and processed the signals for this study [22].

**Figure 2:**
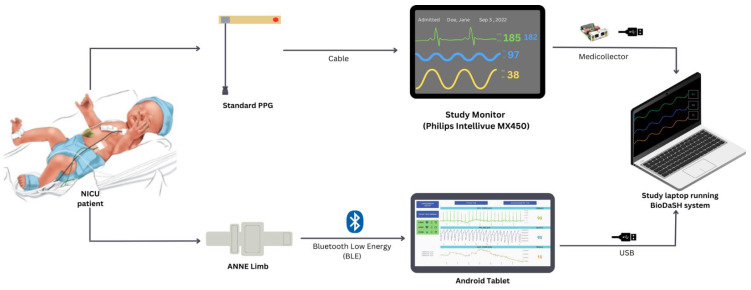
Study design. Legend: NICU: neonatal intensive care unit; PPG: photoplethysmography; USB: Universal Serial Bus. Simultaneous recordings of both systems were possible using Biosensor Data Aggregation and Synchronization (BioDAsh).

Both the standard monitor and the tablet relayed data to a separate BioDAsh system running on a Windows PC. The PC concurrently acquired and stored data from the Philips monitor using MediCollector software (MediCollector, Boston, MA, USA) and from the tablet via USB (Figure 2). BioDAsh also supported live annotation during recordings. All collected data were stored locally on the PC and subsequently uploaded to a secure Dropbox folder using a custom-built Exam Transfer Tool (ETT).

At the end of each study day, BioDAsh recordings were transferred to Dropbox and backed up in parquet file format on an external hard drive. Infants were monitored for eight hours daily across four consecutive days without disrupting clinical care. A research team member remained at the bedside throughout to record annotations and ensure no interference with standard procedures. Demographic and clinical information—including date of birth, chronological and gestational ages, postmenstrual age (PMA), birth weight, current weight, and diagnoses—was collected at enrollment using a standardized data form (see Supplementary Document S1).

### 2.5. Signal Pre-Processing

All signals underwent pre-processing prior to analysis using MATLAB R2023b (MathWorks, Natick, MA, USA). A custom algorithm resampled the signals to a common, uniform rate (64 Hz for PPG and 1 Hz for SpO_2_), and time synchronized the signals from the wired and wireless devices [22]. No data was interpolated in the event that PPG or SpO_2_ signals were unavailable.

### 2.6. Data Analysis

Feasibility was evaluated by analyzing signal coverage and the characteristics of gaps in both the PPG and SpO_2_ signals. Signal coverage was defined as the percentage of the total monitoring period during which data were continuously available. A gap was classified as any instance of missing data—specifically, durations exceeding 31.25 milliseconds for PPG and 2 s for SpO_2_. Gap length distributions were computed for both the wired and wireless systems.

To explore the origin of signal gaps, three categories of variables were examined: (1) automatic alert flags generated by the wireless system indicating Bluetooth connection status and sensor–skin contact integrity, (2) manual annotations recorded by the study team (see Supplementary Table S1), and (3) the signal-to-noise ratio (SNR) of the PPG signals (see Supplementary Figure S1). Each gap was then categorized into one of four possible causes using a decision tree outlined in Supplementary Figure S2. These categories were: (1) Bluetooth disconnections (BD), (2) sensor or lead removals/adjustments (SR/SA), (3) low SNR in the PPG signal (defined as SNR < 0 dB, indicating noise exceeded the true signal), and (4) unknown causes.

Safety assessments focused solely on the wireless device, as it was deemed unethical to remove the standard pulse oximeter purely for research purposes. Skin condition before and after sensor use was documented using photographs taken with an iPad (9th generation, Apple) equipped with an 8-megapixel camera. All images were anonymized, and a board-certified dermatologist—blinded to study details—rated skin condition using the Neonatal Skin Condition Score (NSCS), which ranges from 3 (intact) to 9 (very poor) [23].

To evaluate accuracy, paired SpO_2_ values from the wired and wireless sensors were compared on a sample-to-sample basis. All sample pairs where wired and wireless values were available were included in analysis. Agreement was quantified using Bland–Altman analysis, reporting both the mean bias and 95% limits of agreement (LoA) [24,25]. In addition, mean absolute error (MAE) and margin of error (MoE) were calculated to provide direction-independent estimates of measurement error. Relationships between SpO_2_ error and PPG SNR were visualized using bivariate heatmaps of MAE and MoE, stratified by SNR from both devices.

To assess the clinical impact of discrepancies between the two sensors, a modified Clarke Error Grid Analysis (EGA) was performed separately for infants breathing room air and those receiving supplemental oxygen. Grid boundaries were defined according to the Oxygenate with Love (OWL) protocol’s alarm threshold recommendations, which specify SpO_2_ targets >91% for infants not on oxygen and between >88% and ≤95% for those on supplemental O_2_. Infants were classified as receiving supplemental oxygen if their FiO_2_ exceeded 0.21 for more than 50% of the monitoring period. Table 1 outlines the modified EGA region definitions applied in this analysis.

### 2.7. Sample Size and Statistical Analysis

A convenience sample of 24 neonates was selected, as there was no prior information available regarding the variability in wireless PPG and SpO_2_ signals. To assess feasibility, signal coverage was calculated separately for the wired and wireless PPG and SpO_2_ signals for each recording day (*n* = 96). The resulting coverage distributions were compared using the Wilcoxon signed-rank test, with a significance threshold set at α = 0.001. Gap duration distributions for both systems were visualized using histograms and statistically compared using the Wilcoxon rank-sum test. For gap origin analysis, the proportion of total gaps attributed to each of the four predefined mechanisms was reported. Additionally, cumulative probability distributions of PPG signal-to-noise ratio (SNR) values for the wired and wireless systems were compared using a Wilcoxon rank-sum test.

For the safety evaluation, the Neonatal Skin Condition Scores (NSCS) before and after sensor application were compared using a paired Wilcoxon signed-rank test. In the accuracy analysis, Bland–Altman metrics (bias and limits of agreement) were calculated for each daily recording, and their medians and interquartile ranges (IQRs) were reported. Corresponding effect sizes were calculated, with statistical significance evaluated at α = 0.05 and power set at 90%. For the Error Grid Analysis (EGA), the proportion of sample pairs falling into each defined region of the grid was computed and summarized.

## 3. Results

**Figure 3:**
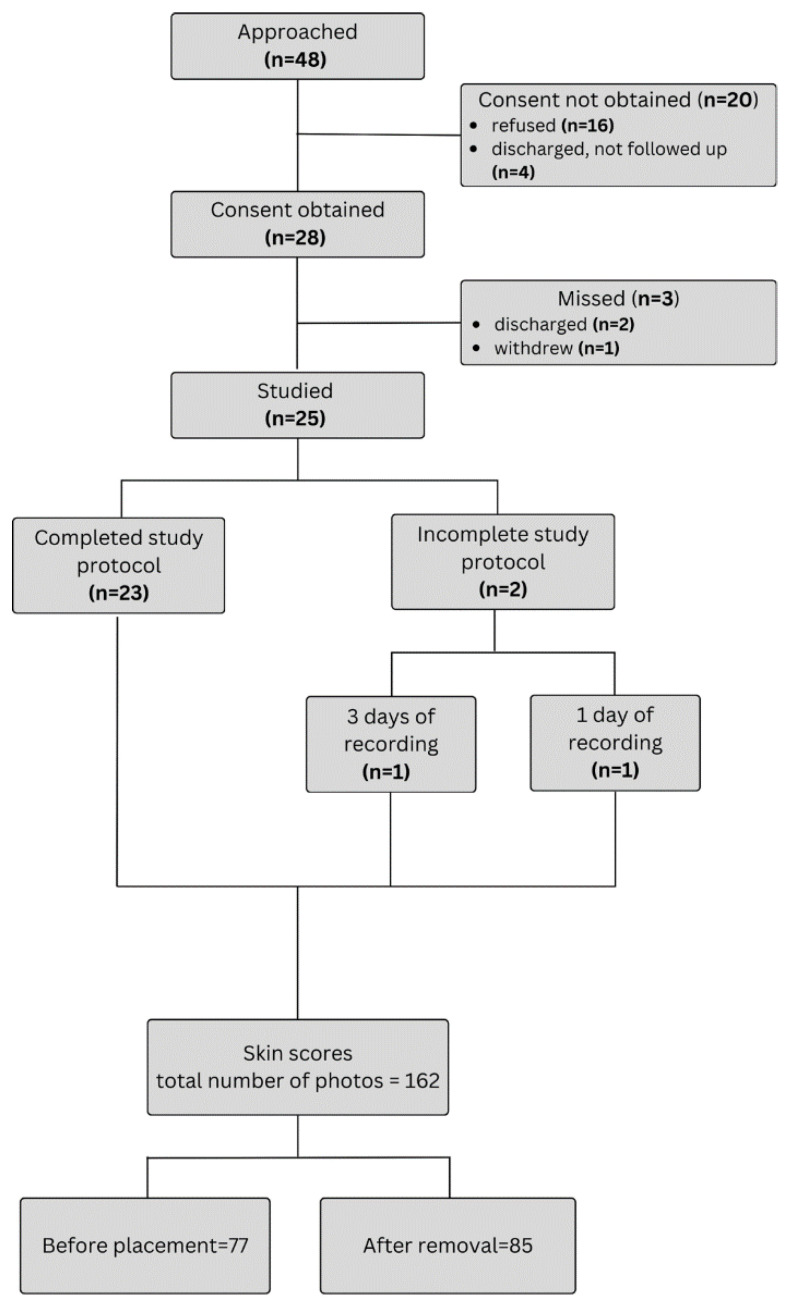
Patient recruitment flowchart.

**Figure 4:**
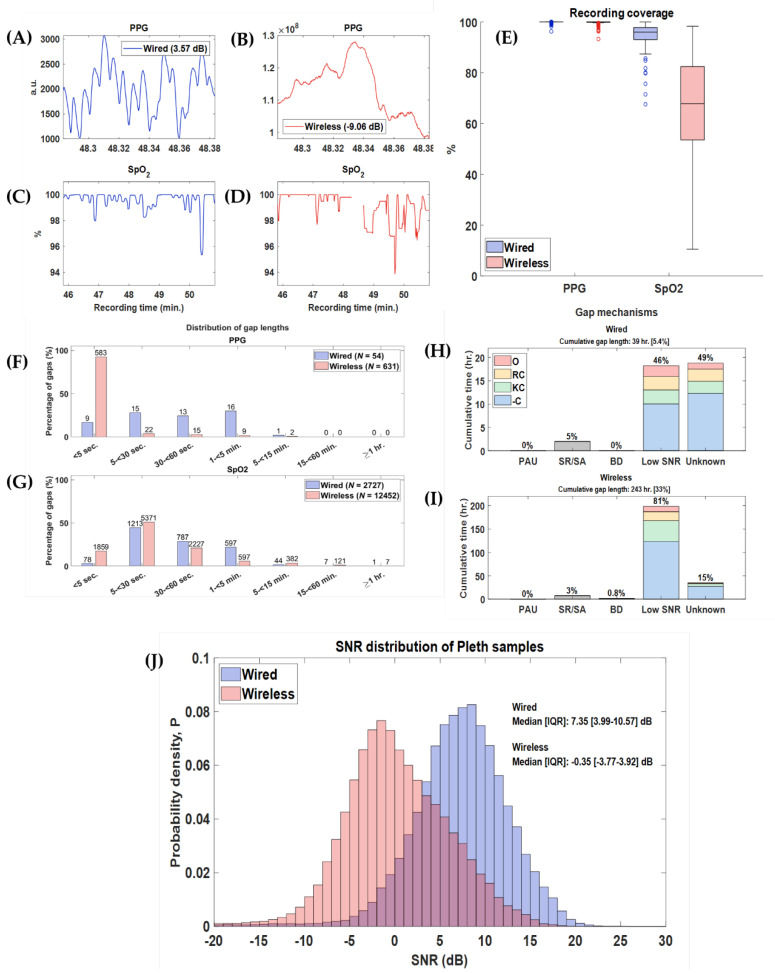
Feasibility analysis. Legend: (**A**–**D**) Example segments of PPG and SpO_2_ signals from a representative patient recording. The wired PPG had an estimated SNR of 10.31 dB with no missing SpO_2_ values, while the wireless PPG showed an SNR of −9.06 dB and contained visible gaps in the SpO_2_ signal. (**E**) Boxplot showing sample coverage for PPG and SpO_2_ across all recordings. (**F**–**G**) Distributions of gap lengths for all PPG and SpO_2_ recordings, with total gap counts labeled above each bar for both the wired and wireless systems. (**H**–**I**) Gaps were attributed to known mechanisms: PAU (pause), SR/SA (sensor removed or adjusted), BD (Bluetooth disconnection), -C (no comment), KC (kangaroo care), RC (routine care), and O (other). These gap mechanisms were further intersected with annotated care activities. The cumulative gap length reflects the total duration during which no signal was available over the 757 h of recording (percentage of total recording time shown in brackets), and percentages displayed above each bar indicate the proportion of cumulative gap time attributable to each mechanism. (**J**) Cumulative probability distributions of SNR values calculated from ECG samples across all recordings.

Recordings were collected over 96 days from a total of 25 infants. One additional participant was enrolled to compensate for four missed recording days: one infant discontinued participation after a single 8 h session, and another withdrew after three days (24 h total) of monitoring (see Figure 3). Some daily sessions were shorter than the intended 8 h due to necessary interruptions for clinical procedures that required sensor removal. In total, 757 h of simultaneous recordings from both monitoring systems were acquired, representing 99% of the planned recording time. Segments of typical wired and wireless PPG and SpO_2_ recordings are shown in Figure 4A–D, additionally typical PPG and SpO_2_ eight-hour recordings, with annotated care activities are provided in Supplementary Figure S3.

**Figure 5:**
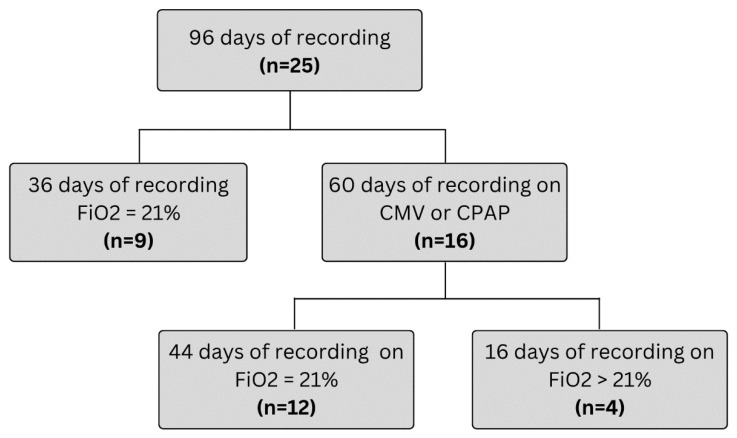
Flowchart of infants on oxygen. Legend: Flowchart of patient respiratory support status; fraction of inspired oxygen (FiO_2_), conventional mechanical ventilation, continuous positive airway pressure (CPAP). Supplemental oxygen is FiO_2_ > 21%.

During recordings, 13 infants (52%) received nasal continuous positive airway pressure (CPAP), 3 (12%) were on mechanical ventilation, and 9 (36%) were stable in room air. Of the 16 patients on respiratory support, 4 received FiO_2_ > 0.21, accounting for a total of 15 days of recordings on supplemental oxygen (Figure 5). Patient characteristics are summarized in Table 2. Table 3 provides information about the participants’ diagnoses at enrollment.

### 3.1. Feasibility

Coverage—The median (IQR) PPG coverage for the wired was 99.96% (99.95–99.96%) and for the wireless signals was 100% (99.85–100%) (*p*-value = 0.97). The median (IQR) SpO_2_ coverage for the wired signals was 95.99% (93.05–97.78%) and 67.79 (53.53–82.41%) for the wireless (*p*-value < 0.01) (Figure 4E).

Gaps—Both the PPG and SpO_2_ signals from the wired system had fewer interruptions than their wireless counterparts: 54 vs. 631, and 2727 vs. 12,452, respectively. Thus, of the 757 h of recording, the wired had 0.01 h (0.11%) of missing PPG data and 26 h (4%) of missing SpO_2_; in contrast, the wireless PPG had 0.05 h (0.44%) and SpO_2_ 254 h (34%) of missing data. The median gap length for the PPG was 0.55 s for the wired and 30.33 s for the wireless (*p*-value < 0.01). For SpO_2_ the median gap length was 31 s for the wired and 22 s for wireless, respectively (*p*-value < 0.01). The majority of gaps in the wireless PPG signal were <5 s (*n* = 92%). Gaps in the wireless SpO_2_ signal were longer, but the majority remained under 30 s (68%). Figure 4F,G shows the distributions of gap length.

Source of Gaps—For the wired PPG signals, all gaps resulted from cable disconnections. In contrast, for the wireless PPG, all gaps were due to Bluetooth disconnections or BioDAsh malfunctions (i.e., disconnection from study laptop and/or unexpected app crash). Figure 4H summarizes the causes of gaps in the wired SpO_2_ signal system; 49% of the gaps were of unknown cause but 46% were due to low PPG SNR, often during care activities. In contrast, Figure 4I shows that the majority of wireless SpO_2_ gaps were due to low PPG SNR (81%), most of which were not associated with any annotated activity (62%). The median SNR in the wired PPG recordings: 7.35 dB [IQR: 3.99–10.57] was significantly greater than the wireless vs.−0.35 dB [IQR −3.77 to −3.92] (*p*-value < 0.01) (Figure 4J).

### 3.2. Safety

A total of 188/200 (94%) skin photographs were taken during the study; 8 (4%) were not taken due to early withdrawal from the study for reasons unrelated to the wireless limb sensor; 4 (2%) were missed by the researcher. Furthermore, 26/188 (14%) photos could not be scored because of low quality or poor visibility of skin. This left a total of 162 photos (77 pre-placement and 85 post-placement) available. Of these, 73 pairs of before-and-after photos were available for comparative analysis.

**Figure 6:**
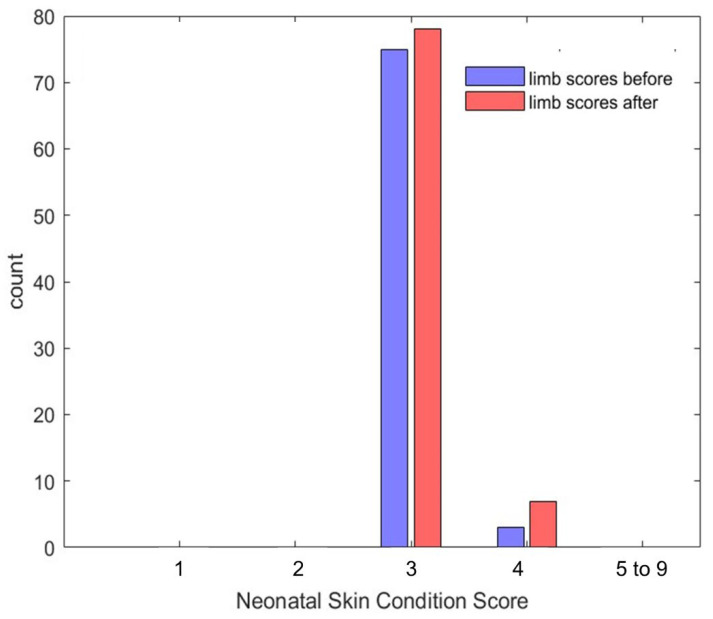
Skin scores. Legend. Distribution of all patients’ scores across all days of recordings, before (blue) and after sensors removal. No statistical difference between before and after scores were observed (*p* = 0.13).

Prior to device placement, all infants had scores of 3 (*n* = 74, 96%) or 4 (*n* = 3, 4%; following sensors removal scores remained in the ranges of 3 (*n* = 78, 92%) or 4 (*n* = 7, 8%) (See Figure 6, Table 4). When comparing the changes between before and after scores, no difference was observed in 63 (82%) photo pairs, an increase by one point was observed in 7 (10%), and a decrease by one point in 3 (4%). A Wilcoxon signed rank test indicated that there was no statistically significant change in the skin scores following device removal (z = −1.12, *p* = 0.13). Detailed skin scores and the distribution of differences are delineated in Table 4.

### 3.3. Accuracy

**Figure 7:**
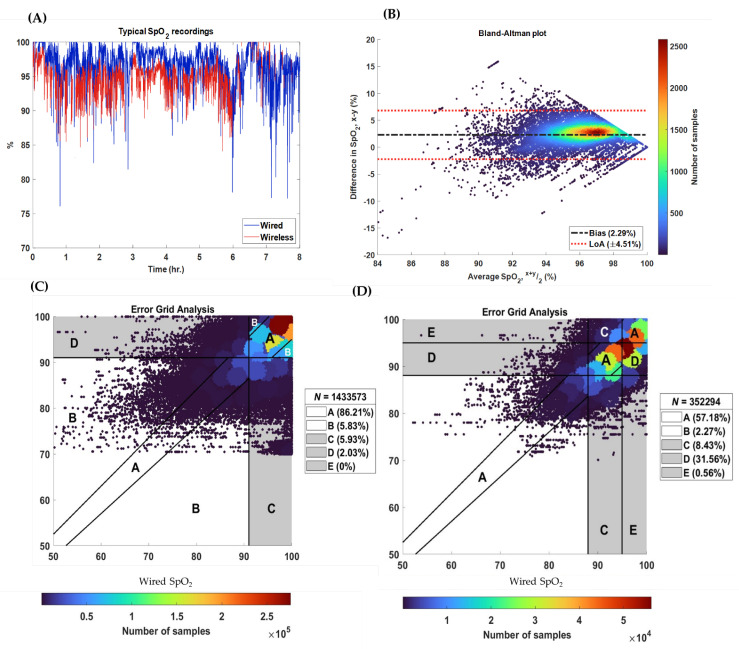
Accuracy analysis. Legend: (**A**) Example of wired (*x*-axis) and wireless (*y*-axis) SpO_2_ signals from a representative patient recording. (**B**) Bland–Altman plot for the corresponding pair of SpO_2_ recordings shown in panel (**A**). (**C**) Modified Error Grid Analysis (EGA) for infants breathing room air, with the percentage of sample pairs located in regions A through E shown in the legend to the right. Region A includes paired values within 5% that would lead to the same clinical decision. Region B includes values differing by more than 5% but still resulting in the same clinical outcome. Region C represents values that would result in unnecessary treatment (i.e., false positives), Region D includes values that would result in failure to treat (i.e., false negatives), and Region E reflects values that would lead to an opposite treatment recommendation. (**D**) Modified EGA for infants receiving supplemental oxygen, with region definitions and legend formatting identical to panel (**C**).

Table 5 provides the median, interquartile range (IQR), and effect size for each agreement metric computed across all 96 paired days of wired and wireless SpO_2_ recordings, and their respective effect sizes. Figure 7A,B demonstrates how these statistical measures were estimated for a typical recording. Bland–Altman analysis revealed a median bias of 1.34 [LoA = −3.62 to 6.41], indicating SpO_2_ values from the wireless sensor tended to be slightly lower than those provided by the wired reference (Table 5). No significant association was found between MAE and MoE and SNR (see Supplementary Figure S4).

Figure 7C presents the modified EGA for all recordings of infants without supplemental O_2_ (*n* = 85 days); 86% of all sample pairs fell within regions A and B, indicating that SpO_2_ values from the wired and wireless sensors would have the same clinical interpretation. Figure 7D presents the EGA for recordings from infants on supplemental oxygen (*n* = 15 days); 57% of the sample pairs fell within region A and B. Importantly 32% of samples fell within region D, indicating the wireless sensor failed to identify a clinically high or low SpO_2_ value (i.e., false negatives). The wireless system SpO_2_ values were systematically lower than the wired values and so tended to miss clinically high values.

## 4. Discussion

This study presents a rigorous framework for evaluating the performance of novel wireless sensors to monitor vital signs under real-world NICU conditions using a wireless SpO_2_ sensor:

(1) Recruitment and detailed characterization of a diverse patient population should be included, and data should be collected using a custom platform that enabled time-synchronized recording of vital signs from both the wired and wireless monitoring devices.
(2) Device feasibility should be evaluated by assessing signal coverage across a range of patient activities and analyzing the impact of routine care tasks on device performance. Following this, safety should be assessed through a formal comparative analysis of the neonatal skin after prolonged device wear.
(3) Accuracy must be evaluated on a sample-to-sample basis and further analyzed using the Clarke error grid to provide clinically meaningful insights.

Key considerations for designing and reporting a comprehensive testing framework are summarized in Table 6.

### 4.1. Comparison to Existing Studies

There are only a limited number of studies exploring the performance of wireless SpO_2_ sensors in hospitalized infants [18,19,20,26,27,28,29,30,31]. This study represents the largest dataset of recordings of patient monitoring with a wireless pulse oximeter, enabled by long and repeated data collection on a variety of NICU patients. Most previous studies have used shorter recordings or focused on healthy term neonates rather than the diverse NICU population [19,20,27,28,29,30,31]. By including a broad range of infants—preterm, term, extremely low birthweight, and those on various respiratory supports, performance was evaluated in clinically relevant, real-world NICU conditions. This contrasts with previous studies where data was analyzed only under optimal conditions, when infants were still and not subject to caregiving activities. Our framework included all recorded data, during routine care and handling [18,19,20,27,31]. This comprehensive approach enhances the robustness of the findings by capturing variations across different NICU scenarios.

Another important issue is that few studies of wireless SpO_2_ sensors have assessed feasibility, often excluding poor signal quality segments without investigating causes, and therefore limiting understanding of device performance. Currently, only three studies have explicitly evaluated feasibility metrics such as signal quality and coverage. Swamy et al. examined “success rate,” a metric comparable to signal coverage, but this was assessed by recordings done within 10 min after birth in the delivery room [31]. Thomas et al. reported the proportion of total recorded data that exhibited poor signal quality over one-hour NICU recordings. Ginsburg et al. evaluated the same sensor used in this study in a Kenyan Maternity Hospital and reported an “up time”—the percentage of the recording for which signal was available and met predefined signal quality criteria—of 69% over 503 h, a finding similar to the 64% coverage observed in our study. Notably, by using our framework, our study is the first to conduct a formal analysis of factors contributing to reduced signal coverage and quality, specifically examining associations with routine patient activities. Poor SNR was identified as the primary cause of signal gaps, highlighting the importance of incorporating such analyses into study designs to inform future developments and enhance device performance. Despite that, the underlying causes of reduced signal quality remain unclear and warrant further investigation. Future studies should incorporate additional measurements, such as precise tracking of device placement, accelerometer-based movement tracking, and measurement of ambient light levels, to better identify potential contributors.

Safety considerations have rarely been explored in studies examining wireless neonatal oximeters despite known risks such as heat-related skin burns and pressure sores. Evaluating skin injury risk and neonatal discomfort is essential. To date, only one study testing a wireless oximeter, has assessed neonatal pain in 40 neonates in a “baby wellness clinic” using the Neonatal Infant Pain Scale (NIPS) [26]. However, skin changes and local temperature increases at the application site, were not formally reported. Our study is the first to conduct a formal analysis of skin safety, demonstrating that the wireless device was safe, though a direct comparison to the standard of care was not possible. Future studies should routinely incorporate safety outcomes, as they are critical for clinical implementation. Additionally, comparisons to existing bedside standard pulse oximeters should be explored, integrating both skin and pain assessments.

Accuracy has been studied most frequently. However, many studies have limitations, including recording durations of an hour or less [19,20,31]. Additionally, several studies restricted recordings to optimal conditions or omitted periods with poor signal quality and/or motion artifacts from accuracy analyses [19,20,27,31]. The present study provides a more granular, sample-to-sample accuracy analysis than other previous studies, which averaged accuracy over intervals from five-minutes to one-hour s, potentially missing important details [26,30]. Three studies reported bias on the device used in this study ranging from 0.11% to 2.65%, with margins of error between ±5.84% and ±6.89% [18,27,28]. The present study, based on a larger dataset, falls within this range but notably found a smaller margin of error (±4.83%), emphasizing the device’s consistent performance over extended recording periods. However, although this margin is smaller, the FDA recommends a root mean square error of ±3%. Exceeding this benchmark has important clinical implications: even modest error margins may obscure sub-optimal SpO_2_ values outside of the targeted thresholds. These potentially missed hypoxic or hyperoxic events can have significant impacts on clinical decision making and infant health. Oxygen therapy is maintained within specific SpO_2_ ranges to avoid both under- and over-supplementation; excess error may transiently expose infants to low or high oxygenation. Such exposures increase the risk of complications associated with inappropriate oxygen levels, including retinopathy of prematurity, bronchopulmonary dysplasia, and adverse neurodevelopmental outcomes.

Beyond overall accuracy, this study also examined the clinical implications of agreement, a factor previously explored in only one study [28]. Ginsburg et al. raised concerns about the device’s ability to detect low SpO_2_ values but this study was not conducted primarily in a NICU setting and excluded infants on respiratory support. For infants on room air, the device detected low SpO_2_ values well. The present study is the first to evaluate clinical accuracy in NICU infants on supplemental O_2_ and our findings reveal challenges in detecting hyperoxia. It should be noted that the sample size for infants on supplemental oxygen was smaller than that for infants on room air, thus limiting the strength of these findings.

The results of this study highlight key areas for improvement and further investigation using the proposed framework. The device demonstrated reliable Bluetooth connectivity and a favorable safety profile. However, future work must address remaining challenges related to accuracy, particularly those stemming from poor-quality PPG signals and the device’s limited ability to detect hyperoxia.

### 4.2. Limitations

This study has several limitations. Recruiting acutely ill infants, such as extremely premature neonates receiving invasive ventilation, was challenging, with many parents declining participation due to concerns about additional equipment and potential increases in disturbances or interventions. As a result, our sample size for evaluating device accuracy in infants at highest risk for severe hypoxia and/or requiring supplemental oxygen was small, which may limit the generalizability of our findings and introduce bias. Future studies should aim to identify and address these barriers to enrollment, for example by reducing study burden through shorter recording durations or minimizing the amount of study equipment, to facilitate participation of these high-risk populations.

Additionally, safety metrics were not measured for the standard wired oximeter, limiting direct comparison with standard care. However, this prospective study provides a comprehensive evaluation of a wireless skin sensor for PPG and SpO_2_ monitoring, with extended recordings conducted during routine NICU care across diverse neonatal populations.

A customized data acquisition system enabled simultaneous sample-to-sample comparisons between wired and wireless oximeters, eliminating the need for indirect methods like video recordings, manual synchronization, or signal averaging [19,26,30]. Unlike previous studies, all recorded data, including segments with infant movement and handling, were analyzed, enhancing the study’s real-world applicability [18,19,20,27,28].

## 5. Conclusions

In conclusion, the framework proposed in this study serves as methodological model to be used for the design and reporting of studies evaluating wireless sensors for monitoring in NICU patients.

## Figures and Tables

**Table 1:** Description of regions in modified EGA.

Region	Interpretation
A	Values within 5% of the reference device and yielding the same clinical outcomes
B	Values greater than 5% of the reference device but still yielding the same clinical outcomes
C	Values that would result in unnecessary treatment (i.e., false positives)
D	Values that would result in failure-to-treat (i.e., false negatives)
E	Values that would result in the reverse treatment

**Table 2:** Patient demographics. Legend: Results are reported as median [IQR] and (min-max). Continuous positive airway pressure (CPAP), and conventional mechanical ventilation (CMV).

	All (*n* = 25)	CPAP (*n* = 13)	Room Air (*n* = 9)	CMV (*n* = 3)
Gestational Age (weeks)	28.4 [26.1–30.7] (25–40.7)	28.4 [26.6–29.3] (25.3–32)	30.7 [28.3–35.8] (26.1–40.7)	25.6 [25.1–26.0] (25–26.1)
Corrected gestational age (weeks)	33.3 [31.3–36.1] (26.3–45.9)	33.1 [31.2–33.8] (26.3–36.7)	36.3 [35.1–40.8] (32.1–45.9)	27.3 [27.0–29.1] (26.9–29.7)
Birthweight (g)	1110 [780–1397] (600–3480)	1200 [806–1356] (600–1790)	1360 [795–2095] (605–3480)	780 [728–1020] (710–1100)
Current weight (g)	1450 [1151–1930] (750–3990)	1450 [1225–1795] (810–2685)	1870 [1378–3028] (1150–3990)	1000 [813–1030] (750–1040)

**Table 3:** Active diagnosis at enrollment. Legend: Results are presented as *n* (%); some participants had more than one diagnosis.

	Infants Studied *n* = 25
Apneas and Bradycardia events	10 (40)
Anemia	8 (32)
Hyperbilirubinemia	8 (32)
Respiratory Distress Syndrome	8 (32)
Intraventricular Hemorrhage	5 (20)
Lung Immaturity	4 (16)
Intrauterine Growth Restriction	2 (8)
Patent Ductus Arteriosus	2 (8)
Bronchopulmonary Dysplasia	1 (4)
Cholestasis	1 (4)
Feeding Intolerance	1 (4)
Gastric Perforation w/ileostomy	1 (4)
Gastro-Esophageal Reflux	1 (4)
Suspected Neonatal Sepsis	1 (4)
Urinary Tract Infection	1 (4)

**Table 4:** Skin safety results. Legend: Results are presented as *n* (%).

**Neonatal Skin Condition Score** (**NSCS**)					
	**Time/Score**	**3**	**4**	**5–9**	**Sum**
**Day 1**	Before	18 (11)	1 (<1)	0	19
	After	22 (14)	1 (<1)	0	23
**Day 2**	Before	20 (12)	0 (0)	0	20
	After	20 (12)	1 (<1)	0	21
**Day 3**	Before	18 (11)	1 (<1)	0	19
	After	19 (12)	2 (1)	0	21
**Day 4**	Before	18 (11)	1 (1)	0	19
	After	17 (11)	3 (2)	0	20
	**Total**	152 (94)	10 (6)	0	162 (100)
∆**NSCS** (**After-Before**)					
	**−1**	**0**		**1**	**Sum**
**Day 1**	1	17		1	19
**Day 2**	0	18		1	19
**Day 3**	1	14		2	17
**Day 4**	1	14		3	18
**Total**	3	63		7	73 (100)

**Table 5:** Accuracy results. Legend: beats per minute (bpm), interquartile range (IQR).

Metric (%)	Median [IQR]	Effect Size
Bias, $\bar{d}$	1.34 [0.53 to 2.20]	±0.64
Margin of error	4.83 [4.05 to 5.96]	±1.19
Upper 95%-limit of agreement	6.41 [5.2 to 7.77]	
Lower 95%-limit of agreement	−3.63 [−4.82 to −2.59]	
Mean absolute error	2.2 [1.18 to 2.93]	±0.5

**Table 6:** Recommendations for future studies with wireless monitoring devices.

Study Component	Recommendations
Population	Diverse, representative NICU population Consideration of adapting the framework to reduce study burden for more acute patients, for example by shortening recording durations, minimizing study equipment, or implementing flexible recording schemes integrated with routine care procedures. Consideration of relevant planned sub analysis in recruitment goals and strategy (i.e., racial, prematurity, supplemental oxygen, events) Clearly defined inclusion and exclusion criteria, with justification
Data acquisition	Extended representative recording duration including routine NICU activities Minimize modification of NICU environment Annotate/document activities during recording Rigorous documentation of relevant variables (i.e., sensor placement, movement, ambient light) Obtain user feedback from parents and HCP
Analysis	● Access to unprocessed raw signals from both devices ● Consideration of averaging schemes of both devices ● A priori defined benchmarks or thresholds for outcomes (i.e., 0 dB considered low quality) ● Explore multiple key outcomes related: feasibility, safety, accuracy ○ Feasibility—frequency of interruptions, context of interruptions, length of interruptions ○ Accuracy—investigate systematic and random error with a priori set thresholds ○ Safety—investigate impacts on skin, with possibility of reference measurement for skin impact ● Consideration of feasibility, safety, and accuracy outcomes in clinical context
Reporting	Full description of study population (GA, PMA, BW, CW, diagnoses) Clear statement of underlying sensing principal (i.e., transmittance photoplethysmography) Clear stating of all pre-processing including synchronization of signals and any interpolation Clear statement of how much data was collected, and subsequently included in analysis (w/explanation of any exclusions) Explicit reporting of any/all adverse events

## Data Availability

The data used in the study are not publicly available because they contain information that could compromise research participant privacy. Anonymized data that support the findings of this study are available from the corresponding author, G.S, upon reasonable request.

## Supplementary Materials

The following supporting information can be downloaded at: https://www.mdpi.com/article/10.3390/s25185647/s1, Supplementary Document S1: Data collection form, Supplementary Table S1: Annotation codes, Supplementary Figure S1: SNR algorithm, Supplementary Figure S2: Analysis Flowchart, Supplementary Figure S3: Typical eight hour signal recording, Supplementary Figure S4: Heatmap analysis.

## Abbreviations

The following abbreviations are used in this manuscript:

NICU	Neonatal intensive care unit
SpO_2_	Oxygen saturation
PPG	Photoplethysmography
FiO_2_	Fraction of inspired oxygen
BioDAsh	Biosensor data aggregation and synchronization system
GA	Gestational age
PMA	Postmenstrual age
DoL	Days of life
BW	Birthweight
CW	Current weight
dB	Decibels
EGA	Error grid analysis
CPAP	Continuous positive airway pressure
SNR	Signal to noise ratio
NSCS	Neonatal skin condition score
LoA	Limits of agreement
MoE	Margin of error
