# Supplementary material for: A Framework to Evaluate Feasibility, Safety, and Accuracy of Wireless Sensors in the Neonatal Intensive Care Unit: Oxygen Saturation Monitoring
Source: Sensors (Basel). 2025 Sep 10;25(18):5647. doi: 10.3390/s25185647 (PMC12473557; doi:10.3390/s25185647)
Supplement: Supplementary file 1 [file sensors-25-05647-s001.zip › sensors-3801394-supplementary.pdf]

Supplementary S1 – DCF

DATA COLLECTION FORM (PHASE 1)

BASIC STUDY INFORMATION

Patient ID: _____________
Date and time of study initiation: _____________ (YYYY-MM-DD) ____________ (HH:MM)
Date and time of study completion: _____________ (YYYY-MM-DD) ____________ (HH:MM)

DEMOGRAPHICS

Year of birth: _______ Sex: ☐ Male ☐ Female Hours of life (at enrollment): ________

Gestational age (at birth): ______ weeks ______ days Birth weight: _________ grams

Gestational age (corrected): ______ weeks ______ days

Diagnosis(es) at time of enrollment:

☐ Perinatal asphyxia ☐ Anemia ☐ Apneas and bradycardias ☐ Bronchopulmonary dysplasia

☐ Hydrocephalus ☐ Intraventricular hemorrhage ☐ Jaundice ☐ Necrotizing enterocolitis

☐ Other, specify: ______________________________

Treatments at enrollment:

☐ Therapeutic hypothermia ☐ Continuous positive airway pressure (CPAP)

☐ Conventional mechanical ventilation (CMV) ☐ High frequency ventilation (HFV)

☐ Nasal intermittent positive pressure ventilation (NIPPV) ☐ Not applicable/healthy infants

Patient group: ☐ A ☐ B ☐ C ☐ D ☐ E ☐ F ☐ G ☐ H

A = healthy term infants in room air at enrollment
B = term infants with perinatal asphyxia undergoing therapeutic hypothermia at enrollment
C = healthy preterm infants in room air at enrollment
D = preterm infants on continuous positive airway pressure at enrollment
E = extremely preterm infants on conventional mechanical ventilation at enrollment
F = extremely preterm infants on high frequency ventilation at enrollment
G = extremely preterm infants on nasal intermittent positive end expiratory pressure at enrollment
H = extremely preterm infants on continuous positive airway pressure at enrollment

Supplementary Table S1 – Annotation Codes

CODE	Reason
AF	Biodash Application Failure/Malfunction
CP-ECG	Change of ECG probes
CP-RR	Change in respiratory probes
CP-SpO2	Change of SpO2 probe
CP-T	Change of temperature probe
PS	Poor Sensor Signal
SR-adj	Sensor readjustment
SR-rep	Sensor replacement
-SX	Sensor removal
B	Bathing
BT	Blood Test (via heel stick or other local acute extraction method)
C	Crying – inconsistent
DC	Diaper change
KC	Kangaroo care
PP	Prone position
SP	Supine position
SS	Side position
RC	Routine Care
S	Sleeping
BF	Breastfeeding
FB	Feeding by bottle
G	Gavage
AC	Airway care
U	Ultrasound
XR	X-Ray
EXAM	10 min
PT	Phototherapy
I	Intubation
E	Extubation
CS	Clinical seizure
O	Other

Supplementary Figure S1 - SNR estimation flowchart for PPG signals

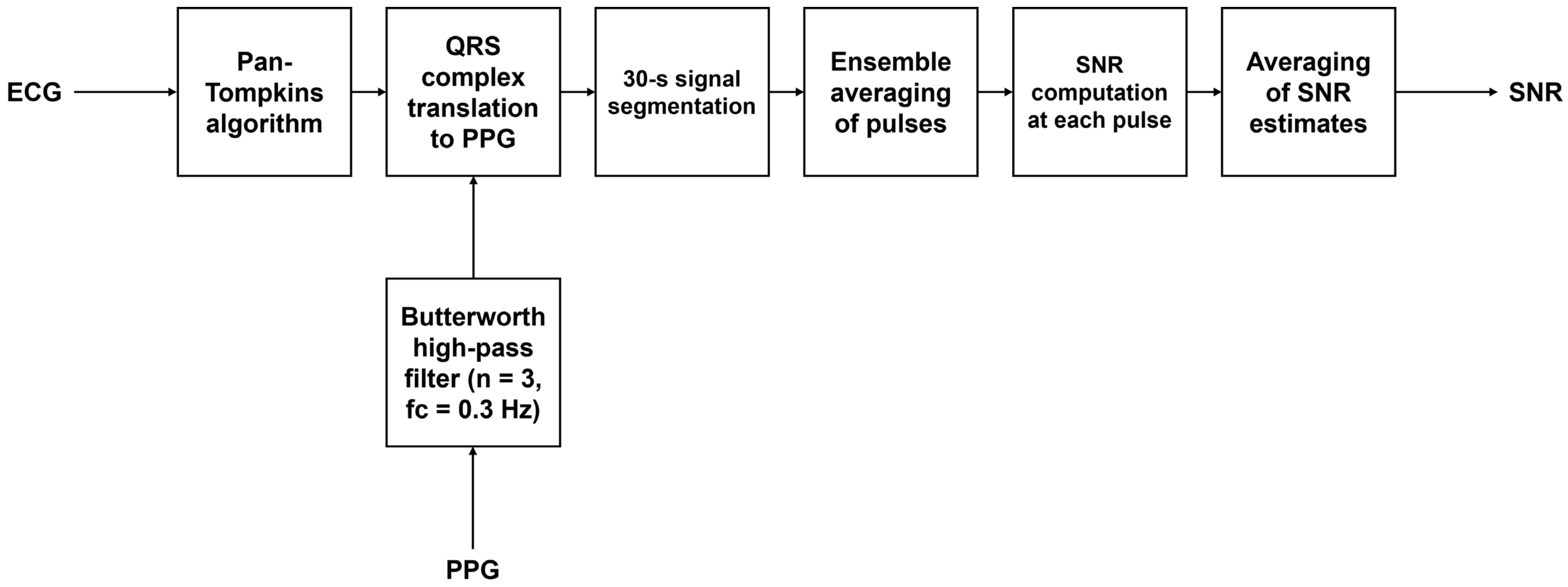

Legend: The algorithm implements ensemble averaging to attenuate additive noise and isolate the desired signal component in the PPG. SNR estimation proceeds as follows: 1. The Pan-Tompkins algorithm is applied to an electrocardiogram (ECG) record to identify all QRS complexes. 2. The corresponding photoplethysmogram (PPG) record is high-pass filtered using a 3rd-order Butterworth filter with a cut-off frequency of 0.3 Hz, to remove baseline wandering. 3. QRS complexes identified in the ECG are translated to their respective positions in the PPG record. The respective locations in the PPG are updated to coincide with the local maximum (peak) of each pulse. 4. The PPG is segmented into 30-s contiguous epochs. 5. In each epoch, all identified pulses contained within the epoch are averaged to attenuate additive noise; the resultant average is taken as the desired signal component (S). 6. The signal component S is subtracted from each of the constituent pulses included in the averaging, to isolate the noise (N) in each individual pulse. The ratio between the variance in S and the variance in N at each pulse is then computed and converted to a decibel (dB) scale; these are referred to as signal-to-noise ratios (SNRs). Finally, the ensemble of SNR estimates in the epoch are averaged to obtain a single SNR value for the entire epoch.

Supplementary Figure S2 – Analysis Flowchart

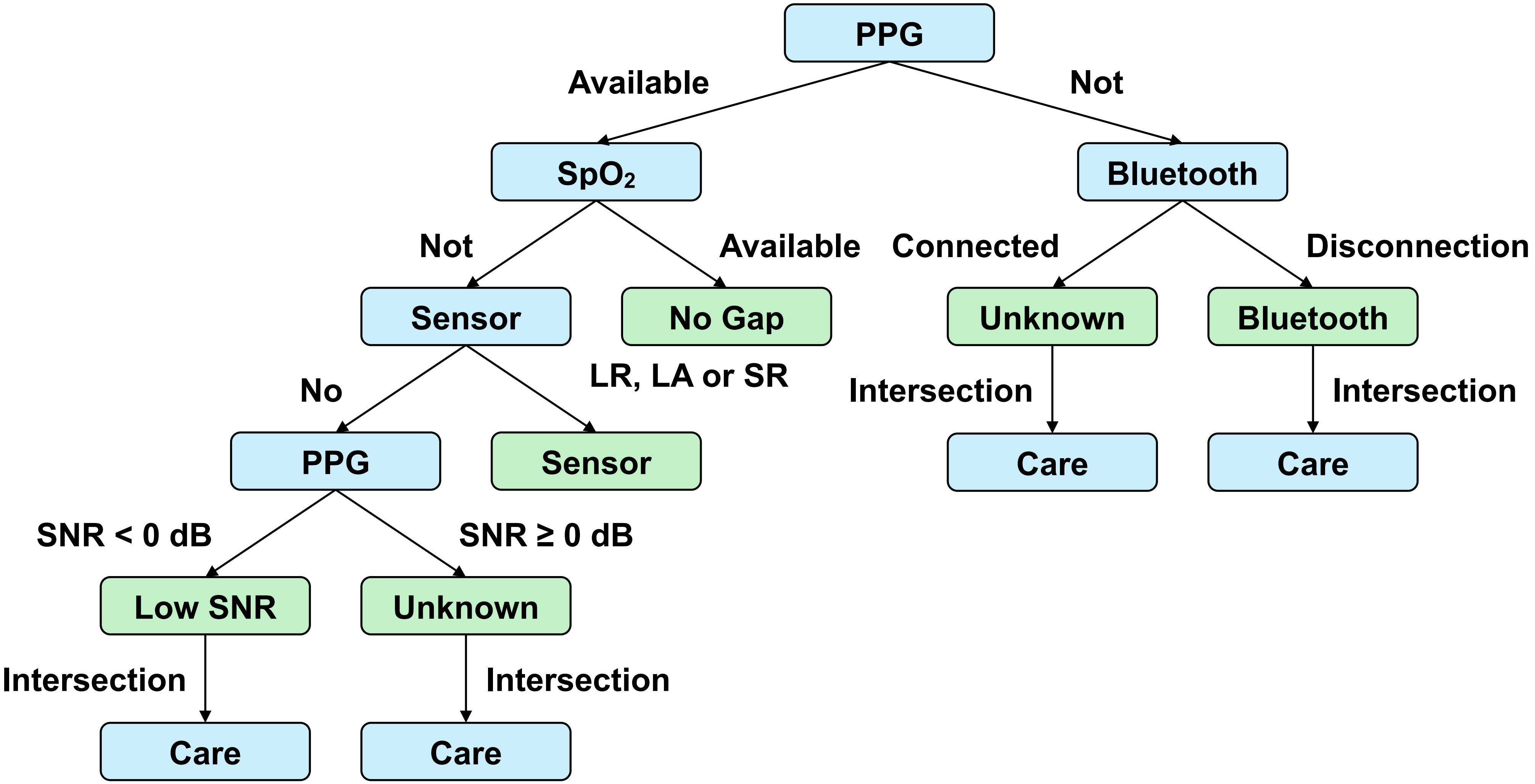

Supplementary Figure S3 – Typical 8 hour recording

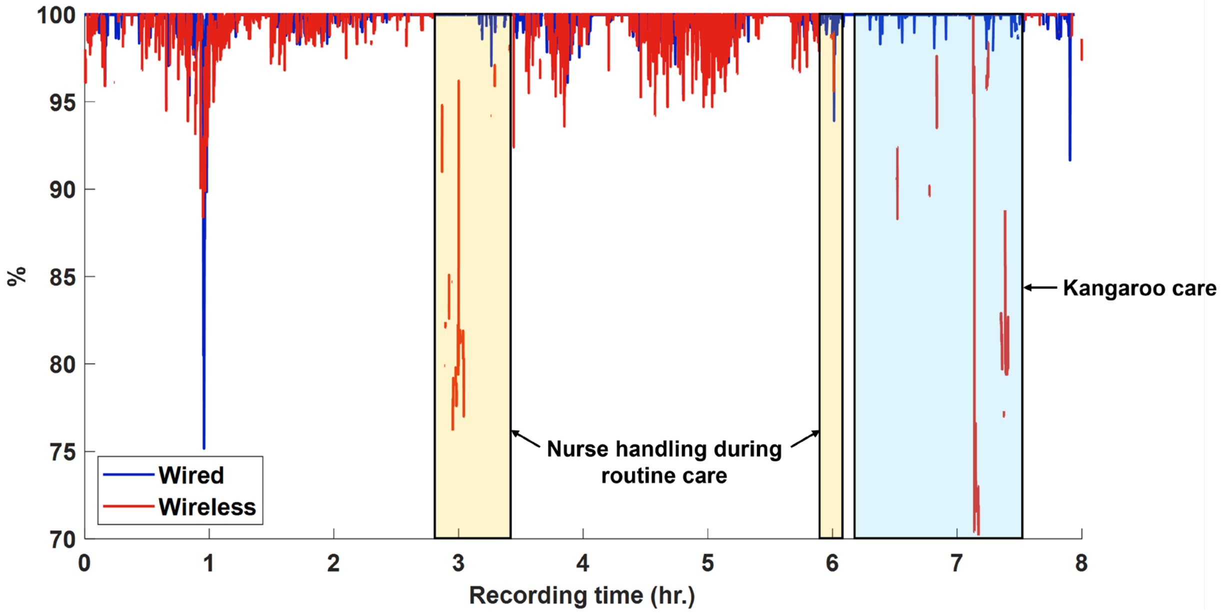

Supplementary Figure S4 – Heat Maps

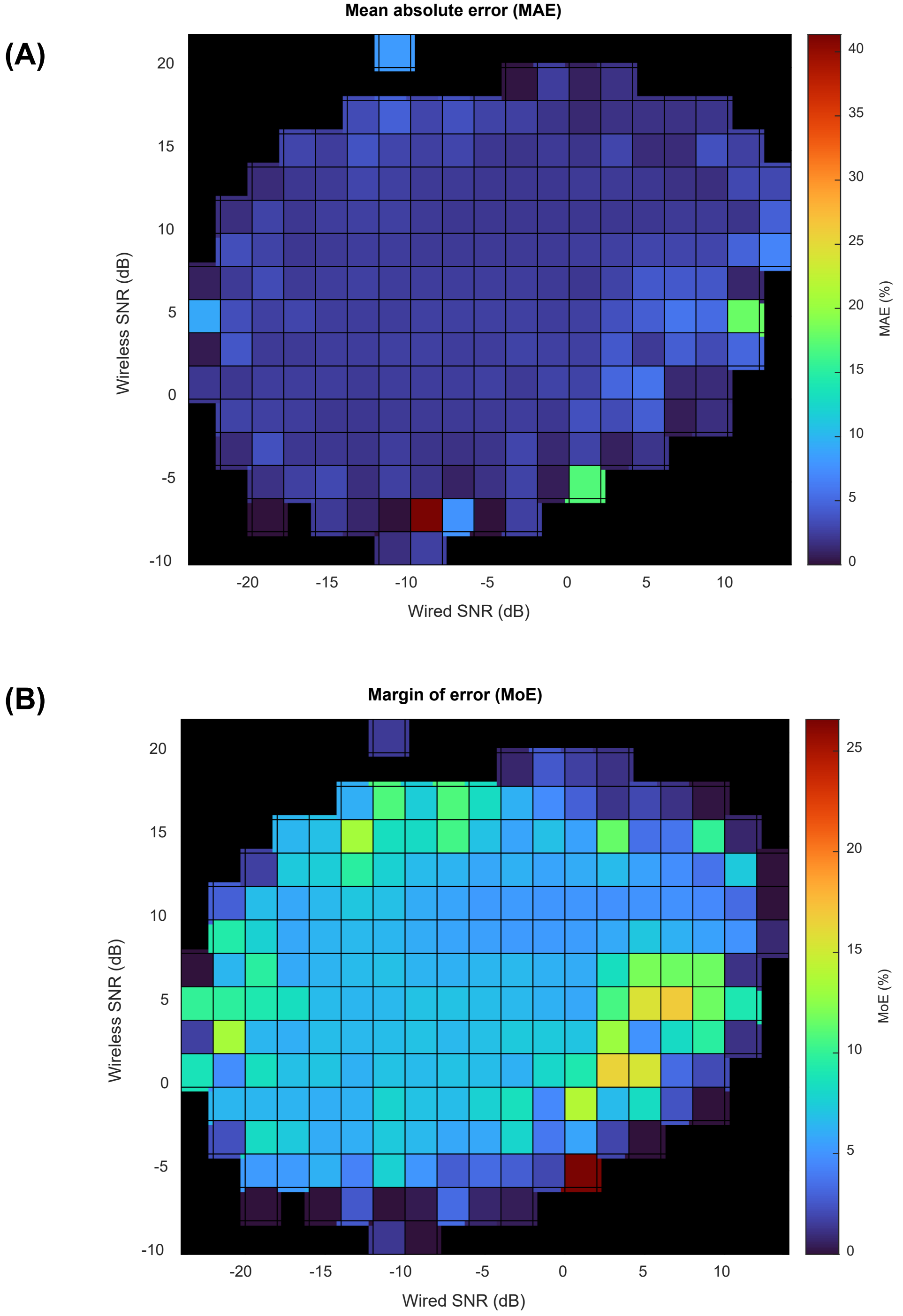

Bivariate heatmaps for (A) MAE and (B) MoE as a function of wired and wireless SNR in the PPG recordings.
